# Exploring drivers for public engagement in social media communication with medical social influencers in China

**DOI:** 10.1371/journal.pone.0240303

**Published:** 2020-10-07

**Authors:** Cindy Sing Bik Ngai, Rita Gill Singh, Wenze Lu

**Affiliations:** 1 Department of Chinese and Bilingual Studies, The Hong Kong Polytechnic University, Hong Kong SAR, China; 2 Language Centre, Hong Kong Baptist University, Hong Kong SAR, China; University of Oxford, UNITED KINGDOM

## Abstract

Social networking sites offer an important means for increasing the accessibility and enabling new forms of health communication between the public and medical social influencers (MSIs). MSIs have a social presence and are perceived as a credible source of health-related information. A research gap, however, exists in understanding the communication strategies employed by MSIs and the factors driving the public to engage in health communication with MSIs. This study, therefore, developed a new conceptual framework incorporating health communication, dialogic and interpersonal communication by employing quantitative content analysis to examine public engagement with MSI communication on the largest microblogging site in China, Sina Weibo. The analysis yielded insights into how the usefulness of health-related information provided alongside the interactive dialogue and affective practices played an active role in engaging the public. The public sought health-related information primarily to address issues of concern for well-being and a high level of engagement in terms of online shares, likes, and comments was found. The use of multimedia made the site more appealing, resulting in likes while the expression of emotions by MSIs generated likes and comments. The need to connect with other online users and have a sense of community was reflected in engagement through sharing useful MSI posts by the public. By identifying influential MSIs on social networking sites, health information providers such as organizations and the government can raise awareness of health issues to foster a healthy lifestyle and contribute to better living in the community.

## Introduction

The rapid expansion of Social Networking Sites (SNSs) such as Twitter, Facebook and Sina Weibo, as sub-types in social media [1], has enhanced users’ interaction and resulted in knowledge sharing, learning [2], dissemination of crucial information [3], and relationship building [4]. Kivitz [5] argued that the Internet and social media (including SNSs) “have made new experiences of health and illness possible” (p.223). The public is increasingly relying on SNSs to gain access to medical-related/healthcare/health behaviour information [6–9]. Fox and Duggan [10] found that approximately 72% of Internet users sought health information online in 2012. Health-related and safety topics have been widely posted on Chinese microblog sites [11]. The number of social media users in China is estimated to reach 679.10 million by 2021 [12], and therefore, social media have the potential to offer credible sources of medical information, promote the adoption of healthy lifestyles and in turn, contribute to better living in society. In the following section, the definition of medical social influencers (MSIs) and the important role played by them, as well as how this study fills the research gap are elaborated.

A plethora of studies exist on patients’ health information-seeking behaviour on the Internet and its influence on the patient-doctor relationship as well as online communication between known doctors and patients [13–15]. However, the successful dissemination of health-related information on SNSs depends on effective dialogic communication and interpersonal influence that determine the choice and sharing of information [16], and MSIs who post information on health-related topics and offer advice, are considered as key social influencers (SIs) in disseminating and communicating health information with the public. SIs are defined as people online who are followed by others and from whom they receive advice regarding purchasing products/services, ultimately influencing attitudes and behavior through their credentials [17]. These influencers have a niche and a large follower base while they share their knowledge on social media [18]. As has been found in a large-scale study, over 70 per cent of communication practitioners in the Asia-Pacific region concur that SIs have an impact on the audience’s attitudes via social media and engagement and are viewed as very important in regions such as China [19]. In our study, we define MSIs as those who possess credentials (qualifications of a registered medical degree and a certification) with credible knowledge [20], and those who provide advice or content on health-related topics. MSIs are also regarded as opinion leaders, the latter of whom are viewed as influential members in social network communities [21]. To the best of our knowledge, studies on the public’s interaction with MSIs are lacking.

The growing use of SNS to access health-related information is also observed among Chinese users, especially on Weibo, the most popular microblogging site in China and the main channel for health-information communication [7]. We therefore contend that the driving factors that lead the public to engage in medical conversations/dialogue with MSIs online in China is a topic worthy of research. This is informed by a recognition that MSIs can contribute to the adoption of healthy lifestyles and a healthy society through raising awareness of health issues and sharing such information with the public, who in turn, disseminate this information to others. In China, the public’s demand for health services is far greater than what can be delivered [22], so MSIs offer a new accessible option to deliver health care. It is well-documented in the medical discipline that health information in social media communication has been emphasized [23–25], while public relations studies have addressed the factors affecting social media interaction and public engagement, including the usefulness of content, dialogic communication, interactivity and social presence [4, 26, 27]. Yet a research gap remains in understanding the dialogic and interpersonal communication that emphasizes medical doctor and online public communication, particularly MSI communication and public engagement in China where MSIs have witnessed an unprecedented growth. Therefore, our study aims to empirically examine the social media communication of top MSIs and public engagement on Weibo in China. The distinctive contribution of this study is that we first drew from the literature on dialogic communication [26], interpersonal presence [4, 28], and health communication [23–25] to examine the driving factors of MSI communication and public engagement on Weibo. Subsequently, we developed a conceptual framework to examine the relationship between these factors and the different levels of public engagement in MSI communication.

This paper is structured as follows. It begins with a review of the role performed by social media in dialogic communication prior to the justification for integrating knowledge from dialogic and interpersonal communication with health communication to provide more clarity on the factors leading the public to engage in health-related communication with MSIs on Weibo. Next, a description of how public engagement is exhibited on social media is given. We then delineate the conceptual framework that has informed this study by reviewing the literature on two driving factors/concepts: dialogic and interpersonal communication strategies that might foster public engagement with MSI communication. This is followed by a categorization of these two factors into five dimensions (i.e. usefulness of information, use of the interactive dialogic loop, affectivity, connectivity, and collectivity). Then, the method, consisting of an empirical analysis of real-world data, is elaborated. The paper ends with an interpretation of the findings, implications of the study, limitations and further research areas.

## Development of a conceptual framework for analyzing factors facilitating MSI communication and public engagement

Creating dialogue between organizations and the public is one of the main ways to foster communication and build relationships [26], and this is increasingly done through social media. Since social media enable real-time and two-way communication, it can enhance communication by sharing information and building dialogic relationships [29]. The health care community has increasingly used social media for interactive communication [30]. An increasing number of online users also rely on social media to obtain health-related information. As reported in the Health Care Development Forum of the 2018 V Impact Summit, there was an exponential increase in the number of users on Weibo (verified- and non-verified users) gaining access to health and medical care information, surging from 51,500 in 2016 to 330,000 in 2018 [31]. A report on "Big Data on Health Concern of Chinese People" by TouTiao and Life Times found that the online reading volume of texts on health-related information by the Chinese public reached an unprecedented high of 33.6 billion texts from Oct 2017 to Oct 2018, with a rise of 33.8% in comparison to the same period in the previous year [32].

However, in studies of how social media facilitate health communication between doctors and the public, the main focus is placed on how health information is disseminated and communicated on social media. Despite a growing number of studies on how doctors engage the public in health communication on social media especially SNSs, these studies rarely investigate doctor-public communication and engagement from a communication and relationship-building perspective with a focus on dialogic and interpersonal communication strategies, two elements that are vital to effective social media communication. To fill the research gap, this study aimed at integrating knowledge from dialogic and interpersonal communication with health communication to contribute to our understanding of the factors leading the public to engage in health-related communication, particularly MSI communication on Weibo. Two driving factors with five dimensions that contributed to different levels of public engagement were identified from the literature on dialogic, interpersonal and health communication [4, 6, 23–26].

Public engagement is closely tied to relationship building and dialogue, and is a process that involves the public in the corporation’s activities [33, 34]. Engagement on social media can be exhibited through metrics/actions such as liking, sharing a post or posting a comment [35–38]. We posit that influenced online users are those who engage with MSIs’ posts through liking, commenting on and sharing them [39]. A ‘like’ indicates one’s perception and requires little commitment while a ‘comment’ and ‘share’ require additional actions that necessitate more cognitive effort [37, 40]. In particular, a ‘share’ suggests a higher level of engagement than a ‘like’ and ‘comment’ since the shared post also appears on the user’s profile page [40] and is disseminated to other social media users through the user’s personal network.

In the next section, we discuss in detail the key factors/concepts of this study by drawing on the theory of dialogic communication, which comprises the provision of useful information and use of the interactive dialogic loop. This is followed by an explanation of interpersonal communication strategies, consisting of affectivity, collectivity, and connectivity, which may facilitate public engagement with MSIs.

### Dialogic communication and health communication on social media

Given the importance of dialogic communication in fostering engagement on social media, this study drew on Kent and Taylor’s [26] theory of dialogic communication in which dialogic communication is defined as “any negotiated exchange of ideas and opinions” that is facilitated by mutual understanding and agreement that is rewarding (p.325). Dialogue focuses on “…meaning making, understanding, co-creation of reality, and sympathetic/empathetic interactions” [41, p. 389]. A key aspect underpinning dialogic communication is the provision of useful content of interest to the public [26, 37]. Other studies have confirmed the importance of this [4, 19]. For example, Men et al. [4] confirmed that leading CEOs generated engagement with the public in the form of likes, shares, and comments on Facebook by providing useful information. MacNamara et al. [19] also found that variables that are viewed as most relevant to identifying SMIs are the relevance of discussed topics and the quality of online content.

As suggested in many health communications studies, useful content is a fundamental factor in facilitating health communication. Bravo and Hoffman-Goetz [23] point out that health content can be divided into specialized health-related information and non-specialized information as well as non-health related information. Specialized health-related information includes content themes (e.g. purpose, symptoms, diagnosis, care logistics, procedures, treatment information, etc.), and communication themes (wording that fulfills relational functions of patient-medical staff communication), such as arguments, reassurance, clarification, and threat [25]. This health care information is usually sought by users who are ill and tends to be a personalized service targeting users who wish to obtain advice on handling symptoms, receiving emotional support and preparing for medical appointments [42–44]. The content also includes information intended to raise users’ awareness of specific health issues. For example, Paige et al. [24] conducted a study on how Pinterest was used as a SNS to disseminate educational information about chronic obstructive pulmonary disease (COPD) to women where the aim was to help patients learn self-management behaviors to reduce the occurrence of COPD symptoms. Similarly, Park et al. [36] examined the use of Twitter by American health organizations to promote health messages and public engagement. Apart from this, Bravo and Hoffman-Goetz [23] noted that non-health related information is frequently communicated on SNSs; they examined the influence of a Movember health campaign, primarily being a moustache competition aimed at raising awareness of men’s health on Twitter in the UK, US and Canada, and found that it generated a high number of shares. Synthesizing the literature above, investigating the effect of health content themes on different levels of public engagement on Weibo is a worthwhile endeavor.

### Interactive dialogic loop

The interaction and communication embedded in SNSs affect health behavior [45], and therefore, the creation of the interactive dialogic loop using interactive features [4, 26] is vital on SNSs. Interactivity is defined as “the degree to which a communication technology can create a mediated environment in which participants can communicate (one-to-one, one-to-many, and many-to-many)…and participate in reciprocal message exchanges…” [46, p. 372]. Having an interactive dialogic loop provides a means through which the public can post questions and comments and share them [26, 47]. A variety of interactive features can be utilized, including multimedia (e.g. videos), stay-up-to-date tools (e.g. newsletters, share/tag tools), click-and-choose activities (e.g. quizzes), and comments on content, forums and games [6]. Hashtags allow the public to find relevant shares on a topic and to identify emerging trends in social media conversations [48], and are often used for creating synchronous conversations on Twitter, leading to engagement and interactivity [36]. A previous study on communication has found that the use of hashtags prevails in interactive communication and a positive relationship between leaders’ use of interactive elements and generation of likes, shares, and comments from the public was found [4]. However, a detailed investigation into the impact of interactive features employed by MSIs on public engagement on Weibo is lacking.

### Interpersonal communication and public engagement

Apart from dialogic communication, public engagement is closely associated with interpersonal communication [49] since social media provide the public with more social support by connecting them to MSIs and other users [50]. Through interpersonal communication with MSIs, the public may become more conscious of making healthier lifestyle choices [51]. Johnston [52] further added that engagement is a “dynamic multidimensional relational concept featuring psychological and behavioral attributes of connection, interaction, participation, and involvement, designed to achieve or elicit an outcome at individual, organization, or social levels” (p.19). In other words, engagement on social media is a collective kind of engagement, which is manifested in behavioral (collective action, group participation), cognitive (the importance placed on content) and affective (experiences) forms [52]. In this connection, interpersonal communication strategies, including affectivity, collectivity, and connectivity, as suggested in previous studies may facilitate public engagement in social media communication [4, 28, 53].

#### Affectivity

The affective strategy focuses on emotional expressions and is exhibited through the use of humor and self-disclosure while being associated with the dialogic orientation of empathy [53]. Men et al. [4] highlighted that the provision of important information to the public alongside affective strategies increased public engagement in the form of likes, shares, and comments in CEOs’ Facebook pages. Similarly, Kang [35] noted that the affective or emotional aspect of the interactions between the public and organizations influenced the public’s behavior towards organizations.

We focus on how the public is engaged by MSIs through their affective communication (e.g. emotional expressions, humor and sharing of personal feelings) in their posts. Such affective feelings include empathy, which is crucial in doctor-patient relationships [54]. In addition, positive affectivity, which refers to the activation of a pleasant emotion [55], can be exhibited in feelings of enthusiasm and humor [35].

#### Collectivity

Engagement is concerned with collectivity that includes an orientation towards dialogue, interaction with stakeholders, active participation, collective action by individuals, and the intention to act [52]. Collectivity could be operationalized as a variety of interactive strategies, including quoting others’ posts and making direct reference to the content of others’ posts, asking questions, and complimenting others [4]. Of these, complimenting others was found to be the most frequently used collectivity strategy by corporate leaders in their social media communication [4]. As such, a study exploring the relationship between collectivity strategies and different levels of public engagement is warranted.

#### Connectivity

Lastly, connectivity, a communication strategy aiming to create a sense of community [28], is an important interpersonal aspect of dialogue [53]. Connectivity necessitates being connected to others and corporate leaders who disclose details of corporations’ operations and employ inclusive pronouns (e.g. we, our, us, our company) are likely to build better relationships with stakeholders [28]. Dholakia et al. [56] note that connectivity is “the feeling of being linked to a world outside the specific site” (p.8), and this means that connectivity is seen when users are connected to the MSI. This sense of community could be created through the use of connectivity strategies, such as inclusive pronouns [28], referring to members of the public by their names in posts, and using social communication like greetings in MSI posts [4]. A prior study has suggested that referring to others by their names is the most frequently employed connectivity strategy and is positively associated with public engagement in corporate leader communication [4]. Yet research on the link between connectivity strategies and public engagement with MSI communication is absent.

## Development of research questions

Underpinned by the above studies, this paper advances the study of online health communication by integrating the theory on dialogic and interpersonal communication to investigate: 1) the use of dialogic and interpersonal communication strategies in MSI communication; and 2) the relationship between MSIs’ employment of dialogic and interpersonal communication strategies on Weibo and public engagement at different levels (i.e. comments, likes, and shares). Specifically, we examined the employment and effects of the five dimensions based on the two driving factors (i.e. usefulness of information content and the creation of the interactive dialogic loop subsumed under dialogic communication; and affectivity, collectivity, and connectivity subsumed under interpersonal communication strategies) on public engagement. Therefore, the following research questions were posed:

RQ1: How did the MSIs employ dialogic and interpersonal communication strategies to engage the public on social media?
RQ2: What was the relationship between the five dimensions of communication strategies and different levels of public engagement?

## Methodology

We examined the paradigm of MSI online communication and the effect of dialogic and interpersonal MSI communication on public engagement in a real-world context. The “real-world” data can be very useful when developing a conceptual framework to reveal the impact of dialogic and interpersonal drivers on MSI-public communication [57]. We used a random sampling method to harness 600 MSI posts and their corresponding public responses manually from the Top 20 MSIs on Weibo for quantitative content analysis, a method widely employed in the study of technical communication [58]. Through an in-depth content analysis of MSI communication on social media, we were able to reveal and establish the relationship between the variables in the proposed conceptual framework.

### Sample selection and collection

We examined MSI communication and public engagement on Sina Weibo, a SNS in China. Sina Weibo is the most popular microblogging SNS in China for marketing services and products [59]. According to a report from the Chinese Academy of Sciences in 2012 [60], 70% of microblog users in China rely on microblog sites as their main source of information. To ensure that medical doctors were sampled, we used the keywords of “医生 (doctor)”, “医师 (physician)”, “个人认证 (V-users)” to ascertain the verified doctors on Weibo; “verified users” need to send documents to Weibo to prove their identities. Once approved, Weibo will assign the letter “V” and a yellow badge to their profile pictures for layman recognition. Verified doctors were preferred in our study as they are more influential in the online community than non-verified ones [59]. Given the huge number of verified doctors found on Weibo, a purposive random sampling method was employed. We employed a self-developed python crawler to identify the Top 20 medical doctors based on their number of followers. These top medical doctors online are coined as MSIs. It is their expertise, ease of access, leadership, and social ties that enable them to influence followers [17].

We then scrutinized the number of posts published by the Top 20 MSIs for three months (from 1 March to 31 May 2019) to ensure that they were active communicators online. Although the Top 20 MSIs published 21982 posts in the sample period, the number of posts varied from 185 to 5923 between the MSIs. Please refer to S1 Appendix for the final list of Top 20 MSIs and their posts published in the sample period. To obtain a balanced sample set from these MSIs, we employed the random sampling method to garner an equal number of Weibo posts from each MSI for conducting content analysis.

Unlike the other open platforms (e.g. Twitter), Weibo has the practice of changing its open API from time to time for technical updates. In addition to this, Weibo imposes “restrictions on the API usage rate” [61, p. 597]. Consequently, we employed a manual data collection method [61] to gather MSI posts and the number of comments, likes, and shares for our study. The data collection method complied with the terms and conditions of the website. Due to the development of the complex coding scheme whereby the 19 sub-dimensions were embedded in the five dimensions of MSI communication, the 19 sub-dimensions were manually coded to reveal the use of various strategies in the MSI posts. To allow the exhaustive manual coding of the 19 sub-dimensions, we decided to harvest a reliable sample size to represent the target population (21982 posts). First, we employed the Sample Size Calculator from the Australian Statistics Bureau to calculate the required representative sample size [62]. To be 95% confident that the true value of the estimate will be within 4 percentage points of 0.5 (i.e., 0.46 and 0.54), the required sample size should be 600. This is the number of actual posts needed to achieve the stated level of accuracy [62]. The standard error of 0.02 on the estimate of 0.5 produced a relative standard error of 4%. Next, we used the random integer generator to generate 30 random integers for each MSI to construct the database of 600 posts. For instance, we used the random number generator to generate 30 random integers out of 185 posts published by MSI subject no. 1 in the sample period (see S1 Appendix). Then, we sampled the 30 corresponding posts from the MSI’s Weibo accounts from 1 March to 31 May 2019 (92 days). Subsequently, we collected the 600 posts and the related number of likes, comments, and shares from the Top 20 MSIs’ Weibo account in the sample period.

### Coding scheme

Content analysis was employed to examine the use of dialogic communication and interpersonal communication strategies in the posts collected from the Top 20 MSIs on Weibo. Drawing on previous studies of dialogic communication [4, 6, 26] and health and medical communication [23–25], we investigated the establishment of dialogic communication manifested in MSI communication from two aspects: the usefulness of information content and the creation of the dialogic loop. First, we manually coded the content of the posts to determine if useful medical/health information was included as follows: 1) Health information about care logistics/procedures/treatment; 2) Health information related to psychosocial aspects; 3) Health information about raising awareness; 4) Non-specialized health-related information; and 5) Non-health related information, on a sentence basis. Secondly, we coded the count number of elements which were employed to facilitate the creation of the interactive dialogic loop. These features included: 1) Reply by the MSI to a user’s questions/comments in the user account/the MSI’s thread; 2) Use of hashtags; 3) Use of multimedia (photos, videos, podcasts); and 4) The use of Games/Surveys/Polls/Quizzes clicks.

**Fig 1:**
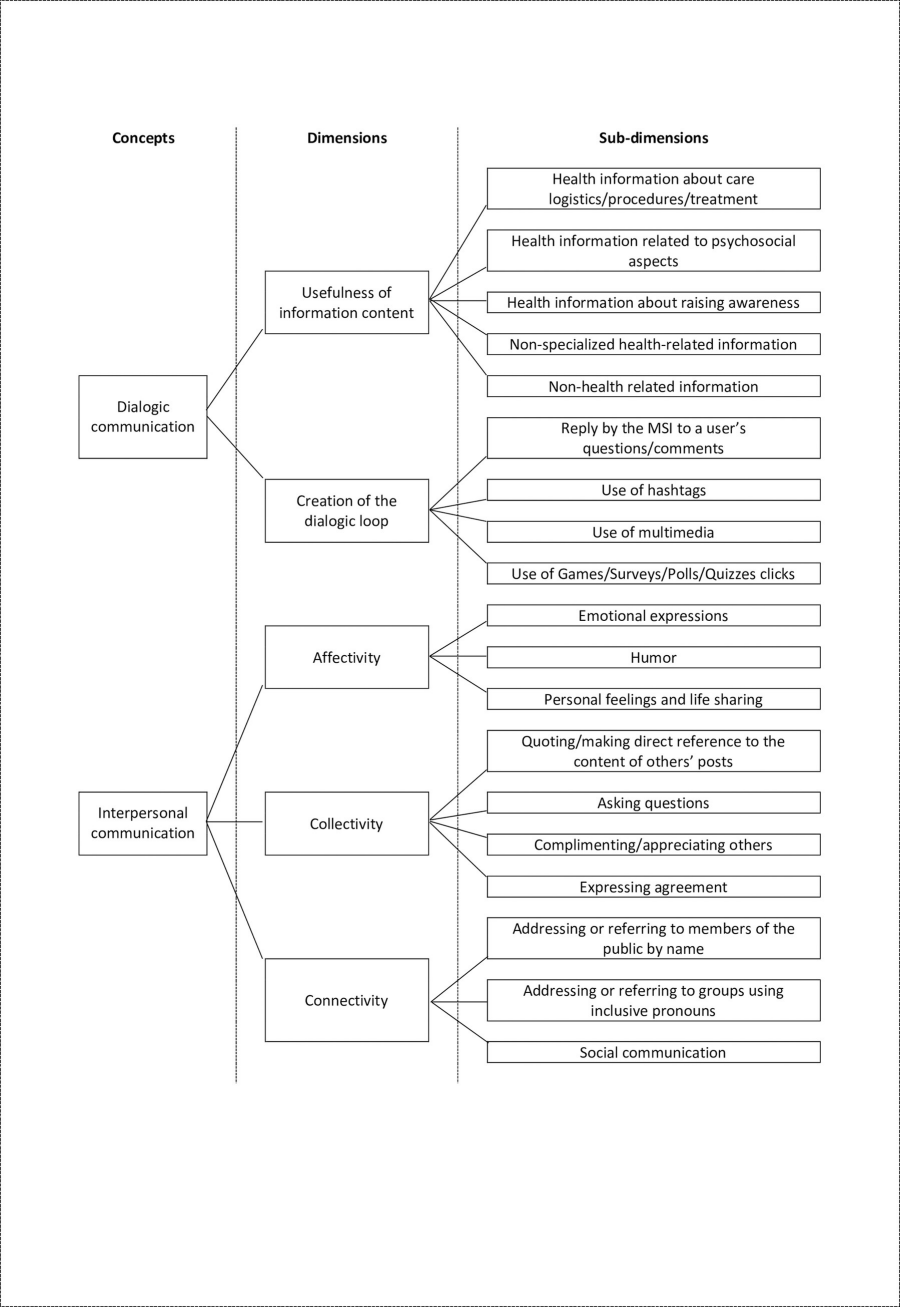
Relationship between concepts, dimensions and sub-dimensions in the integrated framework.

We also built on prior studies [4, 28, 53] and manually coded MSIs’ interpersonal communication strategies according to the three dimensions, namely affectivity, collectivity, and connectivity to reveal how MSIs engaged the public. For affectivity, we coded the count number of: 1) Indicator of emotional expressions (e.g. emotional lexicon, emoticon); 2) Lexical indicators of humor; and 3) Lexical/phrasal verbs indicators expressing personal feelings and life sharing. For collectivity, we coded the sentence number based on the following sub-dimensions: 1) Quoting/making direct reference to the content of others’ posts; 2) Asking questions; 3) Complimenting/appreciating others; and 4) Expressing agreement. Regarding the investigation of connectivity, we coded the count number of: 1) Addressing or referring to members of the public by name (in posts/comments); 2) Addressing or referring to groups using inclusive pronouns (e.g. we, our, us, our group, our community, our society); and 3) Social communication (e.g. greetings, blessings). Please refer to Fig 1 for the relationship between the concepts, dimensions and sub-dimensions in the integrated framework and S2 Appendix for the exemplification of coding items and examples extracted from the 600 collected posts.

To investigate the relationship between MSIs’ employment of dialogic and health communication and interpersonal communication strategies on Weibo and public engagement, we recorded the number of comments, likes, and shares of the 600 sampled posts for statistical analyses.

### Coding procedure and interrater checking

One coder, a full-time research assistant, was comprehensively trained by the second author to undertake half of the coding of posts of MSIs according to the coding scheme. The other half of the coding was completed by the second author. To ensure interrater reliability of the coding of health-related content, interactive dialogic loop, affectivity, collectivity, and connectivity, the coder was repeatedly trained on the coding scheme. The measure of interrater reliability was based on the co-coding of 60 posts of MSIs (10% of the total number of posts studied) by the second author and trained coder. For all categories, the average percent of agreement was greater than 0.97 and the average Cohen’s Kappa was greater than 0.94, indicating an almost perfect agreement [63, 64]. See S3 Appendix for the summary of interrater reliability of the co-coded items.

### Data standardization and statistical analyses

Since the usefulness of information content was coded on a sentence basis, we normalized the coded data by dividing the counted number of sentences by the total number of sentences in each post to yield the percentage of specific information content identified in each post. As for the remaining dimensions, the coding could only be performed in the form of count number of presences.

In relation to RQ1, we tested the data for normality and variance using the Kolmogorov-Smirnov and Shapiro-Wilk test and Levene’s test. As the data showed a violation of assumptions of normality and homogeneity of variance, we performed the Kruskal-Wallis test and post hoc Dunn-Bonferroni adjustment in SPSS to examine the different use of dialogic or interpersonal communication strategies in the sub-dimensions of the five dimensions of MSI communication [65].

Regarding RQ2, given that the dependent variables (i.e. number of comments, likes, and shares) were count outcomes, we employed Poisson regression in SPSS, a count regression model [66] to examine the association between the five dimensions of dialogic and interpersonal communication on public engagement in terms of the number of comments, likes, and shares, as well as the relationship between the sub-dimensions in each dimension and public engagement.

First, we employed SPSS to examine the mean and variance of the count outcomes to determine if there was any violation of assumption in Poisson regression. It is not surprising to uncover a violation of a major assumption in Poisson regression in the real-world dataset [67] involving an overdispersion of outcome variables. In order to improve the goodness of fit of the model, we replaced Poisson regression with Negative binomial regression (NB2) [66]. NB2 is effective in fitting various types of data seen in communication research [68], and “the negative binomial model is a more general model compared with the Poisson regression model that relaxes the strong assumption that the underlying rate of the outcome is the same for each included participant” [66, p.7]. With regards to the examination of the relationship between the sub-dimensions in each dimension and public engagement, a similar practice was adopted as NB2 had largely improved the Goodness of Fit in the conducted tests.

## Findings

### The use of dialogic and interpersonal communication strategies in MSI communication

Pertaining to the use of dialogic and interpersonal communication strategies in the five dimensions of MSI communication examined for RQ1, we found significant differences between the sub-dimensions in all five dimensions measured. For health content communicated, a comparison of health-related information with non-health related information was done. The Kruskal-Wallis test indicated significant differences in the presence of health-related information and non-health related information, H(1) = 55.207, *p*<0.0001, with a mean rank of 530.57 for health-related information and 670.43 for non-health-related information. Then, we examined the employment of different health content themes within the dimension of health-related information. Again, significant differences (H(3) = 204.952, *p*<0.0001) were witnessed between the sub-dimensions. The mean rank for 1) health information about care logistics/procedures/treatment was 1354.71; 2) health information related to psychosocial aspects was 1068.34; 3) health information about raising awareness was 1295.17; and 4) non-specialized health-related information was 1079.34. The post hoc Dunn-Bonferroni test revealed that health information about care logistics, procedures, treatment and health information about raising awareness differed significantly from health information related to psychosocial aspects and non-specialized health-related information. See Table 1 for Pairwise comparisons between the sub-dimensions in the dimension of health-related information.

**Table 1:** Pairwise comparisons between the sub-dimensions in the five dimensions of MSI communication.

Comparing variable	Compared with	Test statistics	SE	Std. test statistics	Sig. (*p*)	Adj. Sig.
**Health-related content**						
Psychosocial	Care logistics, procedures, treatment	286.365	25.172	11.376	0.000	**0.000**
	Non-specialized health info	11.001	25.172	0.437	0.662	1.000
	Raising awareness	-226.829	25.172	-9.011	0.000	**0.000**
Raising awareness	Care logistics, procedures, treatment	59.536	25.151	2.367	0.018	0.108
	Non-specialized health info	-215.828	25.151	-8.581	0.000	**0.000**
Non-specialized health info	Care logistics, procedures, treatment	275.363	25.151	10.948	0.000	**0.000**
**Interactive dialogic features**						
Games/ Surveys/ Polls/ Quizzes	Reply by MSI	-710.945	35.910	-19.798	0.000	**0.000**
	Use of hashtags	-423.205	35.910	-11.785	0.000	**0.000**
	Use of multimedia	-1016.177	35.910	-28.298	0.000	**0.000**
Use of hashtags	Reply by MSI	287.740	35.910	8.013	0.000	**0.000**
	Use of multimedia	-592.972	35.910	-16.513	0.000	**0.000**
Reply by MSI	Use of multimedia	-305.232	35.910	-8.500	0.000	**0.000**
**Affectivity**						
Humor	Emotional expressions	159.639	19.041	8.384	0.000	**0.000**
	Personal feelings and life sharing	-38.298	19.041	-2.011	0.044	0.133
Personal feelings and life sharing	Emotional expressions	121.341	19.041	6.373	0.000	**0.000**
**Collectivity**						
Expressing agreement	Asking questions	312.661	26.983	11.587	0.000	**0.000**
	Complimenting/ appreciating others	6.446	26.983	0.239	0.811	1.000
	Quoting/ making reference to others' posts	-403.810	26.983	-14.965	0.000	**0.000**
Complimenting/ appreciating others	Asking questions	306.215	26.983	11.348	0.000	**0.000**
	Quoting/ making reference to others' posts	-397.364	26.983	-14.726	0.000	**0.000**
Asking questions	Quoting/ making reference to others' posts	-91.149	26.983	-3.378	0.001	**0.004**
**Connectivity**						
Social communication	Addressing or referring to groups using inclusive pronouns	26.044	17.514	1.487	0.137	0.411
	Addressing or referring to members in the public by name	229.421	17.514	13.099	0.000	**0.000**
Addressing or referring to groups using inclusive pronouns	Addressing or referring to members in the public by name	-203.377	17.514	-11.612	0.000	**0.000**

Bold font denotes the significant p value at 0.05.

Concerning the interactive dialogic loop dimension, significant differences (H(3) = 870.36, *p*<0.0001) between the sub-dimensions were found. The mean rank of 1) reply by the MSI to a user’s questions/comments in the user account/the MSI’s thread was 1373.86; 2) the use of hashtags was 1086.12; 3) the use of multimedia was 1679.10; and 4) the use of games/surveys/polls/quizzes was 662.92. The post hoc Dunn-Bonferroni test indicated that the use of multimedia and reply by MSI to users’ questions or comments in the user account or MSI’s post thread differed significantly from the use of hashtags and use of games/surveys/polls/quizzes. See Table 1 for Pairwise comparisons between the sub-dimensions in the interactive dialogic loop dimension.

For the dimension of affectivity, significant differences between the sub-dimensions were revealed (H(2) = 76.629, *p*<0.0001). The mean rank of 1) emotional expressions was 994.16; 2) humor was 834.53; and 3) personal feelings and life sharing was 872.82. The Dunn-Bonferroni test found that emotional expressions varied significantly from humor and personal feelings and life sharing. See Table 1 for the Pairwise comparisons on the sub-dimensions of affectivity.

As for the dimension of collectivity, significances within group differences were found (H(3) = 357.664, *p*<0.0001). The mean rank of 1) quoting/making direct reference to the content of others’ posts was 1423.58; 2) asking questions was 1332.43; 3) complimenting/appreciating others was 1026.22; and 4) expressing agreement was 1019.77. The Dunn-Bonferroni test showed that quoting others’ posts or making direct reference to the content of others’ posts and asking questions differed significantly from complimenting others and expressing agreement. See Table 1 for the Pairwise comparisons on the sub-dimensions of collectivity.

Significances within group differences were also revealed for the dimension of connectivity (H(2) = 205.767, *p*<0.0001). The mean rank of 1) addressing or referring to members of the public by name was 1044.77; 2) addressing or referring to groups using inclusive pronouns was 841.39; and 3) social communication was 815.35. The post hoc Dunn-Bonferroni test confirmed that addressing or referring to members of the public by names in posts and comments varied significantly from addressing or referring to groups using inclusive pronouns and social communication. See Table 1 for the Pairwise comparisons on the sub-dimensions of connectivity.

### Effect of driving factors in MSI communication on public engagement

In relation to RQ2, which inquired into the effects of the five dimensions of dialogic communication and interpersonal communication strategies on public engagement, we first examined the association between the five dimensions and public engagement in terms of comments, likes, and shares. Next, we investigated the relationship between the sub-dimensions and public engagement.

For the relationship between the five dimensions and public engagement, the coefficient (indicated as B) and the incident rate ratio (indicated as EXP (B)) in Table 1 revealed that health-related information was the best predictor of comments and likes, and particularly shares, while the interactive dialogic loop was positively associated with likes. In other words, for every unit increase in the number of sentences on health-related content, the number of comments, likes, and shares increased by 1.62, 1.59 and 2.31 times respectively. Further, the number of likes would be expected to increase 1.1 times for every unit increase in the features of the interactive dialogic loop. Regarding the interpersonal communication dimensions, affectivity had a positive effect on the number of comments and likes and collectivity had positive effects on the number of comments. For every unit increase in affectivity, the number of comments and likes would be expected to increase 1.12 and 1.18 times respectively, and for every unit increase in collectivity, the number of comments would be expected to increase by a factor of 1.16, while holding all other variables in the model constant. Of the interpersonal communication dimensions, connectivity was the strongest predictor of shares because the number of shares would be expected to have a rate 2.72 times greater for every unit increase in connectivity. See Table 2 for the negative binomial results on the five dimensions and the number of comments, likes, and shares.

**Table 2:** Negative binomial results on the five dimensions and the number of comments, likes, and shares.

Predictor variables	Number of comments						Number of likes						Number of shares					
	B	SE	Sig.	Exp (B)	95% Wald Confidence Interval for Exp(B)		B	SE	Sig.	Exp (B)	95% Wald Confidence Interval for Exp(B)		B	SE	Sig.	Exp (B)	95% Wald Confidence Interval for Exp(B)	
**Health- related content**	0.482	0.153	**0.002**	1.619	1.200	2.184	0.465	0.138	**0.001**	1.591	1.214	2.085	0.838	0.311	**0.007**	2.311	1.256	4.254
**Interactive dialogic loop**	0.028	0.021	0.188	1.028	0.986	1.072	0.044	0.022	**0.047**	1.045	1.001	1.092	0.021	0.025	0.415	1.021	0.972	1.072
**Affectivity**	0.114	0.044	**0.009**	1.121	1.029	1.221	0.163	0.048	**0.001**	1.177	1.071	1.293	0.035	0.065	0.588	1.036	0.912	1.177
**Collectivity**	0.145	0.050	**0.004**	1.156	1.048	1.275	-0.083	0.046	0.072	0.920	0.840	1.007	0.123	0.226	0.587	1.131	0.726	1.762
**Connectivity**	-0.082	0.060	0.176	0.921	0.819	1.037	-0.003	0.577	0.956	0.997	0.890	1.116	1.001	0.217	**0.000**	2.722	1.780	4.163

Bold font denotes the significant p value at 0.05.

### The association between the sub-dimensions and public engagement

Our subsequent examination of the relationship between each dimension in the two clusters of factors and different levels of public engagement found that for the dimension of health-related information, the Negative binomial regression results indicated that both health-related and non-health related content were significantly positively associated with the number of comments but not related to likes and shares. For each extra sentence on health-related content in a MSI post, 3.2 (95% CI, 1.77 to 5.79, *p*<0.001) times more comments were yielded while for every extra sentence on non-health related content, 2.37 (95% CI, 1.32 to 4.25, *p*<0.01) times more comments were found. As for the sub-dimensions in health-related content, the sub-dimension of health information about care logistics/procedures/treatment was positively associated with comments whereas non-specialized health information was positively related to comments and likes. For every extra sentence on health information about care logistics/procedures/treatment, 1.6 times (95% CI, 1.09 to 2.34, *p*<0.05*)* more comments were found. For every extra sentence on non-specialized health information, 4.31 times (95% CI, 1.90 to 9.75, *p*<0.001) more comments and 2.95 times (95% CI, 1.42 to 6.13, *p*<0.01) more likes were generated.

Concerning the sub-dimensions in the interactive dialogic loop, NB2 results revealed that three out of the four sub-dimensions in the interactive dialogic loop had a relationship with likes and shares. For every additional hashtag provided, 2.45 (95% CI, 1.83 to 3.26, *p*<0.0001) times more shares were generated. Furthermore, 1.14 (95% CI, 1.06 to 1.21, *p*<0.001) times more likes were found for every extra feature of multimedia provided. However, for every additional game/survey/poll/quiz included, 0.04 (95% CI, 0.00 to 0.30, *p*<0.01) times fewer likes and 0.01 (95% CI, 0.00 to 0.26, *p*<0.01) times fewer shares were generated.

For the sub-dimensions in interpersonal communication, we noted a variation in the relation between the predictors and outcomes. With regards to the sub-dimension of affectivity, a positive association between emotional expressions and likes was noted. For every extra indicator of emotional expressions, 1.3 (95% CI, 1.09 to 1.55, *p*<0.01) times more likes were generated.

Regarding the sub-dimensions of collectivity, complimenting others was not associated with likes, comments, and shares. However, NB2 results revealed both positive and negative relationships between the remaining three sub-dimensions and likes, comments, and shares. For every additional quote/reference to others’ posts and agreement expressed, 1.18 (95% CI, 1.06 to 1.30, *p*<0.01) and 7.13 (95% CI, 2.88 to 17.68, *p*<0.0001) times more shares were found respectively. Furthermore, for every extra effort in quoting others’ posts/making direct reference to the content of others’ posts, 0.83 (95% CI, 0.76 to 0.90, *p*<0.05) times fewer likes were found, while for every additional agreement expressed, 2.01(95% CI, 1.03 to 3.94, *p*<0.05) times more likes were noted. Lastly, for every extra question asked, 1.21 (95% CI, 1.05 to 1.40, *p*<0.05) times more comments were generated.

As for the sub-dimensions of connectivity, we found 1.41 (95% CI, 1.30 to 1.54, p<0.0001) and 3.88 (95% CI, 1.81 to 8.31, p<0.001) times more shares for every additional reference to others by their names and using social greetings respectively. However, we noted 0.88 (95% CI, 0.82 to 0.95, p<0.01) times fewer likes for every extra reference to others by their names.

## Discussion

### Effects of dialogic and communication strategies in MSI communication on public engagement

The purpose of this paper was to examine how the public is engaged by MSIs on Weibo through developing and empirically testing a conceptual framework underpinned by concepts on health communication, interactive dialogic loop and interpersonal communication. Our results offered consistent evidence that health-related content as opposed to the other dimensions contributes to the highest level of public engagement in terms of the number of likes, comments, and especially shares. Sharing, which is a distinct characteristic of social media, enables users or the public to reproduce the information by sharing it [69] with a large number of users. Thus, sharing as a form of engagement, which enables users to link the MSI post to their social group [70], is vital in disseminating health-related information more widely and rapidly. Additionally, our findings revealed that health-related information about care logistics, procedures and treatment [25] was most frequently observed in MSIs’ posts and positively associated with comments. This is likely because seeking and communicating health-related information is a goal-oriented process and a conscious choice [71] and users would only visit Weibo to seek health information if it is directly relevant to their needs. Since users spend time and effort in seeking health information from MSIs, they would prefer to obtain relevant information only, consistent with Deng et al.’s [72] findings who noted that information quality was of crucial importance in health-seeking behavior through mobile phones. This topic also generated more comments due to the personalized nature of consultation advice provided by MSIs to users. It is also worth noting that non-health related information was positively associated with the number of comments, suggesting that such information is frequently communicated on SNSs [23] while confirming that a large number of microblog users in China rely on Weibo as their key source of information about not only health but other aspects such as personal life and social events [60].

The results also emphasize the importance of engaging users by the interactive features embedded in the interactive dialogic loop in that a positive association was found between the use of this and the number of likes, the latter of which indicates the popularity of posts [73]. Important health information supplemented with photos and videos (i.e. multimedia) can promote dissemination efforts and facilitate the public perception that the information is useful [6, 74], while hashtags enable the public to share important topics in social media conversations [48]. The public also needs to have their questions and comments addressed for dialogue to occur [26], and this is indeed consistent with our findings. It should be noted that although hashtags were positively associated with likes, they were used significantly less than multimedia features and replies by MSIs to users’ questions. Hashtags were under-used because they were not regularly provided by MSIs in China for the public to view specific information by clicking on them [36] on social media.

Notably, the results provide evidence that Weibo users were actively engaged by affectivity, especially emotional expressions employed by MSIs because significant results were found with respect to comments and likes. Affectivity plays a key role in building relationships between corporations and the public [4, 52]. The empathy developed between MSIs and the public involves an understanding of personal feelings and the disclosure of sensitive, personal information, consequently the likelihood of this being shared was lower, although this manner of engagement generated a higher number of likes. Our study found that humor was less likely to be used in MSIs’ posts even though Gough et al. [39] suggested that humorous and shocking messages resulted in higher levels of engagement with skin cancer prevention messages on Twitter. Health content on Weibo encompasses a wide range of areas such as treatment/care logistics and non-specialized information, but humor may not be suited to the dissemination of medical information related to personalized health care, particularly in the Chinese context.

Concerning collectivity, the results revealed that complimenting others in MSI communication was used significantly less than quoting others’ posts/making reference to others’ posts and asking questions. Users are motivated to seek health-related information for a purpose such as resolving a health issue [67], and hence, the use of overly polite expressions such as complimenting others might be inappropriate in this context. Asking questions was linked to public engagement in the form of a higher number of comments possibly because questions posed to MSIs answer a user’s specific health problems but at the same time, promote dialogue and interaction among other users [4, 70].

Regarding engagement as connectivity, referring to others by their names was the salient connectivity strategy employed. In online health communication, a sincere, personalized relationship must be established between the MSI and user, and by using names, the different parties can easily build rapport with each other. A large number of shares was linked to addressing members of the public by their names in posts as well as using social communication, which is partly consistent with Mao and Zhao’s [75] findings that doctors on online medical websites preferred to use salutations or social communication, leading to the projection of a polite image of doctors and earning respect from patients. Overall, the findings suggest that different sub-dimensions of the dialogic and interpersonal communication strategies are effective in MSI engagement with SNS users with respect to generating comments, likes, and shares.

### Theoretical and practical implications

The results add to the body of knowledge on public engagement with health communication on Weibo. First, our developed conceptual framework of public engagement with MSI health communication was empirically tested and we found that the usefulness of health-related information is the strongest driver generating engagement with respect to likes, comments and shares, followed by interactivity, which is linked to likes; affectivity, which is related to likes and comments; collectivity, which is associated with comments; and connectivity with shares. Second, we have gained insights into how seeking health-related information tends to be personally driven by the incentive to address a health-related symptom/concern or take preventive measures, so relevant health information is vital followed by the use of multimedia to make things more interesting, as well as the expression of emotions such as empathy by MSIs concerning health problems. The need to connect with other online users and have a sense of community is exhibited through sharing useful MSI posts and making reference to others’ posts by the public.

Third, this study helps advance research by providing insights into the need to include more health-related information since the public has a strong desire for such information. Finally, the results have implications for health practitioners designing health campaigns to increase the public’s awareness of health issues. They can draw on our findings in making predictions about what the public would find useful when they undertake a search on MSIs and health-related information. It is imperative for health practitioners and researchers to gain a thorough understanding of SNSs in the dissemination of health information and customize the information in accordance with potential health campaigns [76]. By identifying MSIs who are influential on SNSs, health information providers can realize their purposes of disseminating health awareness messages on a large scale at a low marketing cost to the community [16] to promote the adoption of healthy lifestyles. Since social media facilitate the dissemination of information to other users or the public more widely and rapidly [69, 70], while our study has shown that health-related information and the communicative strategy of connectivity foster the sharing of MSI posts, MSIs can mobilize these strategies to reach a larger audience and deliver health care. Because MSIs are a reliable source of health-related information, the public could be persuaded to change their health behavior. Information-seeking behavior of the public influences their decisions to engage in healthy lifestyles and the information provided by MSIs can help make positive changes in the public’s health practices according to scholars [77, 78]. As Bennett and Glasgow [79] note, the World Wide Web can be harnessed to disseminate health information, and thus, MSIs can use Weibo to improve preventive medical care. Prospective MSIs can frame their social media health communication with a sense of empathy, expression of feelings, and relationship orientation, consistent with the key aspects of dialogue in an attempt to attract followers. As Kent and Taylor [26] highlighted, usefulness of information contributes to building dialogue, thereby in a bid to enhance the informative value of content, using videos and photos, relying on emotional expressions, making reference to others’ posts, asking questions, referring to users by their names, and adopting social greetings can encourage the public to continue following a certain MSI.

## Conclusion, limitations and further research

This article contributes to the advancement of dialogic theory and interpersonal communication and extends to Weibo use in a health communication context. It has specifically contributed to our understanding of the importance accorded to useful health-related information, interactivity, affectivity, collectivity, and connectivity by the public on Weibo through developing a framework that operationalized these measures. Armed with this knowledge, researchers and health practitioners can better understand the behavior of SNS users in seeking MSIs’ expertise and their engagement with MSI health communication.

Our conceptual framework on public engagement in online health communication could be tested through experimental research to confirm the validity of the model and strength of the five factors. The inclusion of more sub-categories in the dimensions of affectivity, interactive dialogic loop, collectivity, and connectivity could have generated slightly different findings. This framework can be applied in other cross-cultural contexts to develop a thorough understanding of how MSIs engage the public. Since only the top 20 MSIs on Weibo were examined, this potentially limits the generalizability of the findings. A specific demographic is more likely to use SNSs to seek health information, such as Internet users below age 65, university graduates and those with more online experiences [80–82], so our findings may not be generalized to the entire population. Analyzing the number of likes, comments and shares over a longer timeframe will be helpful in strengthening the study’s findings. Additionally, it is difficult to ensure that the MSIs’ medical qualifications and information are 100% genuine on Weibo. At present, there are no policies regulating MSIs on Weibo other than ascertaining whether their qualifications are recognized, so more regulations are needed to ensure that the public uses the information provided by MSIs appropriately. The public, medical doctors, government, and non-government organizations, industry bodies, and MSIs need to simultaneously address the public’s health needs and concerns. Future research should examine a higher number of MSIs’ engagement with users on different SNSs and one could further refine the sub-categories of the various dimensions to obtain more robust results. Ultimately, since the usefulness of information is viewed as crucial, future studies can examine users’ perceptions of the credibility, relevance, and value of information from MSIs and their willingness to engage in dialogic communication through a qualitative study.

## Supporting information

S1 Appendix List of top 20 MSIs and their posts published in the sample period. (DOCX)

S2 Appendix Exemplification of coding items and examples extracted from the database. (DOCX)

S3 Appendix Summary of inter-rater reliability. (DOCX)

S1 Data (XLSX)
