# Supplementary material for: Exploring drivers for public engagement in social media communication with medical social influencers in China
Source: PLoS One. 2020 Oct 7;15(10):e0240303. doi: 10.1371/journal.pone.0240303 (PMC7540861; doi:10.1371/journal.pone.0240303)
Supplement: S1 Appendix — (DOCX) [file pone.0240303.s001.docx]

**S1 Appendix. List of top 20 MSIs and their posts published in the sample period**

| **Ranking** | **Name of the medical social influencer on Weibo (English translation)** | **Total no. of posts (in 92 days)** | **Average post/ day** |
| --- | --- | --- | --- |
| 1 | 崔玉濤 (Cui Yu Tao) | 185 | 2.010869565 |
| 2 | 蝦米媽咪 (Xia Mi Mommy) | 236 | 2.565217391 |
| 3 | 安定醫院郝醫生 (Anding Hospital Dr. Hao) | 298 | 3.239130435 |
| 4 | 鮑秀蘭診室 (Bao Xiulan Clinic) | 2454 | 26.67391304 |
| 5 | 急診科女超人於鶯 (ER Superwoman Yu Ying) | 212 | 2.304347826 |
| 6 | 龔曉明醫生 (Dr. Gong Xiaoming) | 238 | 2.586956522 |
| 7 | 腫瘤專科醫生 (Oncologist) | 484 | 5.260869565 |
| 8 | 白衣山貓 (Baiyi Shanmao) | 239 | 2.597826087 |
| 9 | 張思萊醫師 (Dr. Zhang Zilai) | 5923 | 64.38043478 |
| 11 | 整形醫生修志夫 (Plastic surgeon Xiu Zhifu) | 942 | 10.23913043 |
| 12 | 甘肅劉維忠 (Gansu Liu Weizhong) | 397 | 4.315217391 |
| 13 | 居家瘦譚小軍老師 (Ju Jia Shou Tan Xiaojun Laoshi) | 502 | 5.456521739 |
| 14 | 心理學劉愛民 (Psychologist Liu Aimin) | 2086 | 22.67391304 |
| 16 | 婦產科醫生王玉玲 (Gynecology Dr. Wang Yuling) | 545 | 5.923913043 |
| 17 | 雙眼皮修復脂肪填充整形-Dr蔣 (Shuang Yan Pi Xiu Fu Zhi Fang Tian Chong Zheng Xing- Dr. Jiang) | 268 | 2.913043478 |
| 18 | 白衣鹹飯 (Baiyi Xian Fan) | 847 | 9.206521739 |
| 19 | 急救醫生賈大成 (ER Dr. Jia Dacheng) | 540 | 5.869565217 |
| 20 | 眼科小超人老梁 (Ophthalmologist superman Lao Liang) | 1474 | 16.02173913 |
| 21 | 整形醫生cici (Plastic surgeon cici) | 3829 | 41.61956522 |
| 22 | 地壇醫院劉彥春 (Di Tan Hospital Liu Yanchun) | 283 | 3.076086957 |
