# Supplementary material for: Exploring drivers for public engagement in social media communication with medical social influencers in China
Source: PLoS One. 2020 Oct 7;15(10):e0240303. doi: 10.1371/journal.pone.0240303 (PMC7540861; doi:10.1371/journal.pone.0240303)
Supplement: S2 Appendix — (DOCX) [file pone.0240303.s002.docx]

**S2 Appendix. Exemplification of coding items and examples extracted from the database**

| **Coding items** | **Examples (with English translation)** |
| --- | --- |
| **Health related information- specialized (HRI-S)** | |
| Health info about care logistics/ procedures/ treatment | No. 1: 10 Apr, 06:09  孩子至今不会走路，应该有神经发育问题。尽快看神经科，寻找原因。至于进食，但体重低，也要结合神经系统发育，排除代谢性疾病。当然，需要家长将孩子整个生长发育过程详细过程呈现给医生。  Literal translation: If the child can't walk so far, he probably has neuro-developmental problems. See the Neurology Department of the hospital as soon as possible to find out the reason. As for a child who eats but is underweight, you need to consider the nervous system development to further exclude metabolic diseases. Parents are required to present the detailed process of the child's growth and development to the doctor. |
| Health info related to psychosocial aspects | No. 1: 18 Mar, 06:23  爱美之心，人皆有之。生完宝宝后，绝大多数妈妈都想尽快恢复体型，皮肤光鲜亮丽等。为此染发，烫头，染指甲等都可以进行。 妈妈会自己掌握分寸的。  Literal translation: Everyone has a desire for beauty. After giving birth to the baby, most mothers want to recover their body shape and bright skin as soon as possible. Thus, hair dyeing, perm, fingernail dyeing can be carried out. A mother should take care of herself. |
| Health info about raising awareness | No. 3 25 Mar, 21:29  美国食品药品监督管理局（FDA）批准了首个针对产后抑郁的临床注射用药Zulresso (brexanolone)，为产后抑郁的个性化诊疗开辟了新道路。注射给药，起效迅速，使用48小时就会开始缓解中到重度抑郁，传统抗抑郁药物2周后才起效；联用其他抗抑郁药不影响疗效；不影响母乳喂养。  Literal translation: The Food and Drug Administration (FDA) approved Zulresso (brexanolone), the first clinical injectable drug for postpartum depression, which opened a new way for personalized diagnosis and treatment of postpartum depression. Such medical drug has rapid effect after being injected in 48 hours (e.g. alleviates moderate to severe depression compared to traditional antidepressants which take effect after 2 weeks; combined with other antidepressants, the efficacy is not affected; does not affect breastfeeding). |
| Heath related information- non-specialized (HRI-NS) | No.2 (pediatrician): 11 Mar, 17:06  为了这一代和下一代，烟盒上要印刷恐怖警示图片且不能陈列在货架明显处卖。可以仔细考虑一下，从烟草行业获得的税收和因为吸烟，二手烟，三手烟导致的健康问题而投入的医疗资源哪个更高!  Literal translation: For this generation and the next generation, cigarette boxes are required to be printed with terror warning pictures and cannot be displayed on noticeable shelves for sale. We can think carefully about the income from the tobacco industry in contrast to the costs of health problems caused by smoking, second-hand smoke and third-hand smoke. |
| Non-health related information (NHRI) | No.2 (pediatrician): 30 Mar, 23:24  好久没见到我的猫咪了，最近经常和朋友夸它脾气好，结果昨天出门前，它在我旅行箱上撒尿，搞得我差点赶不上高铁。  Literal translation: I haven't seen my cat for a long time. Recently, I have often praised him for his good temper in front of my friends. As a result, he peed on my suitcase before going out yesterday, which made me almost unable to catch the high-speed railway. |
| **Interactive Dialogic Loop** | |
| Reply by social influencer to a user’s question/ comments in user’s account/social influencer’s comment thread | No.2: 10 Mar, 20:52  名字已占：三年级了，人教版？  虾米妈咪(No.2)回复 @名字已占：嗯，是湖南出的教材。  名字已占回复 @虾米妈咪：哦，我看课本安排很像人教版，所以问一下。  Literal translation:  *Name already occupied*: “third grade, Peoples Education Edition?”  *Shrimp-rice-mummy (No.2)* replies @*Name already occupied*: “Well, it's a textbook from Hunan, absolutely PEE” .  *Name already occupied reply* @*Shrimp-rice-mummy*: “Oh, I think the textbook arrangement is very similar to PEE, so I asked you to confirm that”. |
| Use of hashtags | No.1: 21 Apr, 06:22  化脓性扁桃体炎只是说明疾病状况，并不是病因，需寻找原因-比如：通过咽分泌物查甲型溶血性链球菌或其他病菌。  确定是细菌感染，才可考虑用抗生素。是否用静脉抗生素，需根据病情。确诊甲型溶血性链球菌感染，青霉素类抗生素至少连用10天。#崔玉涛讲常见病#  Literal translation: Pyogenic tonsillitis can reveal a disease condition, but not identify a cause. We need to find out the cause - for example, through the pharyngeal secretion to detect type A hemolytic streptococcus or other bacteria. Antibiotics should be considered only if bacterial infection is confirmed. The use of intravenous antibiotics depends on the condition of the patient. Penicillin antibiotics were used for at least 10 days in the diagnosis of Streptococcus haemolyticus A infection.  # Cui Yutao talks about common diseases# |
| Use of multimedia (photos, videos, podcasts) | No.3: 29 May, 21:18  【教育心理学】孩子的自信是如何被摧毁的？1、有条件的爱，让孩子心生恐惧；2、苛求完美，让孩子总觉得自己不够好；3、总抱怨牺牲过多，让孩子有罪恶感；4、脾气暴躁，常情绪失控，甚至打骂孩子；5、要求孩子绝对服从；6、凡事包办替代，不相信孩子；7、总是别人家的孩子好；8、在外人面前让孩子出丑。  Literal translation: [Educational Psychology] How is a kid’s self-confidence destroyed?  1. Conditional love, making the child fear;  2. Striving for perfection, resulting in that children always feel they are not good enough;  3. Complaining too much, leading a kid to be full of guilt;  4. Bad temper, uncontrollable emotions and scolding kids;  5. Requiring absolute obedience of children;  6. Everything arranged by parents; no trust in the kids;  7. Always praising other families’ kids;  8. Letting the children make a fool of themselves in front of outsiders. |
| Games/Surveys/ Polls/ Quizzes clicks | No.3: 25 Apr, 22:30  昨晚在评论区里因参与游戏而获奖的三位朋友，注意私信给我联系方式哦。  Literal translation: To the three friends who won a prize for participating in the game in my comment thread last night, please give me the contact information. |
| **Engagement as Affectivity** | |
| Emotional expressions | NO.8: 29 Apr, 23:20  反观昨天，我批评浦东警察铐走医生合法不合情时，一些自媒体却采取给我泼子虚乌有的脏水搞人身攻击。这么低的格局，让我不屑回应。站对不站队，讲理讲法不搞人身攻击，如果连这一点都做不到，个别自媒体的格局就太低了。  Literal translation: On the contrary, when I criticized the Pudong police for handcuffing doctors unreasonably and illegally, some online self-media attacked me with scornful words and a condescending attitude. Such a low pattern makes me unwilling to respond. We need to be rational, resisting personal attacks. If you cannot even do this, the pattern of self-media is too low. |
| Humor | No 2: 15 May, 15:00  受腾讯邀请参加2019中国互联网公益峰会。本以为这个季节飞广州的航班总是无限延误，结果不仅没有延误还提前落地了（emoji: laugh)。大概老天也知道我还没准备好讲什么（emoji: laugh)。  Literal translation: Invited by Tencent to attend the China Internet Public Welfare Summit in 2019, I have thought that flights to Guangzhou were always delayed in this season. However, the flight landed safely ahead of the scheduled time (emoji: laugh). Maybe God knows that I am not ready to make a speech (emoji: laugh). |
| Personal feelings and life sharing | No 2: 3 May, 01:16  昨天儿子跆拳道比赛，我的丈夫当时在国外，我把儿子比赛的视频发给他，告诉他儿子很有攻击力，以后不要打孩子。儿子还跟我说，对爸爸永远不会动手，除非爸爸敢对妈妈动手。就冲儿子这句话，这十多年 辛苦也值得。  Literal translation: Yesterday, my son played Taekwondo while my husband was abroad at that time. I sent him a video of my son's game and told him that his son was very aggressive and warned him not to hit his son in future. My son told me that he would never attack his father unless his father hits and yells at his mother one day. I feel touched and I’m lucky to raise my son. |
| **Engagement as Collectivity** | |
| Quoting others’ post/making direct references to the content of others’ post | No.6: 15 Apr, 23:43  哇靠！我该重修妇产科了。//@东土大唐三俗和尚：博大精深//@地下天鹤绒：中医发展太快了。  Literal translation: Oh my god, It's time for me to review and learn again about gynecology and obstetrics. //@ User1: Broad and Profound //@ User2: The development of traditional Chinese medicine is too fast. |
| Asking questions | No 4: 26 Mar, 09:11  宝宝被蚊子咬了怎么办？用冷水敷还是热水？最简单的办法，叮咬的部位如果没有破溃用肥皂水清洗即可。  Literal translation: What if the baby is bitten by mosquitoes? Is using cold water or hot water better? Actually, the easiest way is to clean the bitten wound with soapy water. |
| Complimenting others, appreciating others | No 3: 16 Apr, 10:17  No 3: 感谢你的分享！  @用户：去年11月份去世的同事Andrew,生前完成了巴黎圣母院的扫描工作，他营造的网站包括教堂各个角度的建筑全景，3D和细节图片-网页链接  Literal translation: No 3: Thank you for sharing!  @ User: My colleague Andrew, who passed away in November last year, completed a scan work of Notre Dame in Paris. His website includes panoramic views of the church from all angles, 3D and detailed pictures - Web links. |
| Expressing agreement | No 4: 10 Mar, 18:46  No 4:你说的确实是个问题，我同意你的额观点，二手烟和三手烟对孩子的危害是很大的。  @User: 只能去劝孩子的爸爸不在孩子面前抽烟，但是衣物上的三手烟是无法避免 的。  Literal translation: *No 4:* what you said is indeed a problem. I agree with you. Second-hand and third-hand smoke do great harm to children, too.  *@User:* I can only persuade the children's father not to smoke in front of their children, but second-hand and third-hand smoke on clothing cannot be avoided. |
| **Engagement as Connectivity** | |
| Addressing or referring to members of the public by their name(s) | No.1: 2 May, 06:14  @朱二曼和冯二浩：医生您好，我儿子两周九个月了，之前排便排尿都挺好的，近期总是尿裤子。说了很多次，打了很多次，还是尿裤子，我该怎么办啊?  Literal translation: @ Zhu Er Man and Feng Er Hao: Hello, Doctor. My son is almost three-year old and had no problem of urinating since birth. However, he has always been getting his pants wet by peeing recently. I scolded him many times sometimes even beat him, with no effect on his behavior at all. What should I do?  崔玉涛：首先说教和暴力不能解决问题，反而增加孩子的抵触情绪！遇到能力倒退现象，首先应该寻找原因，比如：家中二孩出生；家长对孩子管教方式改变或过于粗暴；其他心理问题等。  Literal translation:  Cui Yu tao: First of all, preaching and violence do not solve the problem at all, but increase children's resistance! When we encounter the phenomenon of ability regression, we ought to look for the reasons firstly, such as the second child born in the family, excessive rudeness in the way of children's discipline, and other psychological problems, etc. |
| Addressing or referring to groups using inclusive pronouns | No 3: 26 May, 21:06  比起记忆力，我们更需要忘记的能力。人到中年，如果对很多事情无法释怀会很痛苦。人要学会忘记，才能快乐生活。  Literal translation:  We need the ability to forget rather than to remember. In middle age, it can be painful if you can't let go of many things. Only when people learn to forget can they live happily. |
| Social communication | No 14. 1 Apr. 19:10  《您好，医生，之前我已经跟您咨询过，当时您建议我可以依照您的方法去做，可如今我的情况有所转变，请问现在需要注意什么》—作为医生我的建议是......-网页链接  Literal translation:  《Hello, Doctor, I have consulted with you before. At that time, you suggested I needed to follow your advice, but now my situation has changed a bit. What should I pay attention to now?》As a doctor, my suggestion is ......- Web link. |
