# Supplementary material for: Exploring drivers for public engagement in social media communication with medical social influencers in China
Source: PLoS One. 2020 Oct 7;15(10):e0240303. doi: 10.1371/journal.pone.0240303 (PMC7540861; doi:10.1371/journal.pone.0240303)
Supplement: S3 Appendix — (DOCX) [file pone.0240303.s003.docx]

**S3 Appendix. Summary of inter-rater reliability**

| **Coding Items** | **Percent Agreement** | **Scott's Pi** | **Cohen's Kappa** | **Krippendorff's Alpha** |
| --- | --- | --- | --- | --- |
| Health info about care logistics/procedures/treatment | 91.7% | 0.896 | 0.896 | 0.897 |
| Health info related to psychosocial aspects | 96.7% | 0.855 | 0.855 | 0.856 |
| Health info about raising awareness | 90% | 0.807 | 0.807 | 0.809 |
| Heath related information- non-specialized | 95% | 0.91 | 0.91 | 0.913 |
| Non-health related information | 93.3% | 0.903 | 0.903 | 0.904 |
| Reply by the social influencer to a user’s questions/ comments in user account/the social influencer’s comment sector/thread | 100% | 1 | 1 | 1 |
| Use of hashtags | 100% | 1 | 1 | 1 |
| Use of multimedia | 100% | 1 | 1 | 1 |
| Games/ Surveys/ Polls/Quizzes clicks | 100% | 1 | 1 | 1 |
| Emotional expressions | 93.3% | 0.867 | 0.867 | 0.868 |
| Humor | 100% | 1 | 1 | 1 |
| Personal feelings and life sharing | 95% | 0.845 | 0.845 | 0.846 |
| Quoting others’ post/making direct references to the content of others’ post | 93.3% | 0.899 | 0.899 | 0.9 |
| Asking questions | 100% | 1 | 1 | 1 |
| Complimenting others, appreciating others | 100% | 1 | 1 | 1 |
| Expressing agreement | 100% | 1 | 1 | 1 |
| Addressing or referring to members of the public by name | 96.7% | 0.934 | 0.934 | 0.935 |
| Addressing or referring to groups using inclusive pronouns | 100% | 1 | 1 | 1 |
| Social communication | 100% | 1 | 1 | 1 |
